# Simulation of the Effects of Minimum Case Volume Thresholds on the Geographic Accessibility of Inpatient Neurosurgical Facilities in Germany

**DOI:** 10.3390/healthcare14152437

**Published:** 2026-08-06

**Authors:** Simon Mayer, Stervenson Jean Pierre Licht, Özlem Alarslan, Benjamin Mayer, Dalibor Bockelmann, Benjamin Voellger, Thomas Kapapa

**Affiliations:** 1 Strategic Medical Controlling, Ulm University Hospital, Albert-Einstein-Allee 29, 89081 Ulm, Germany; 2 Department of Neurosurgery, Ulm University Hospital, Albert-Einstein-Allee 23, 89081 Ulm, Germany; 3 Institute of Epidemiology and Medical Biometry, University of Ulm, Schwabstraße 13, 89075 Ulm, Germany; 4 Department of Medical Strategy and Networking, University Medical Centre Freiburg, Breisacher Straße 153, 79110 Freiburg, Germany; 5 Department of Neurosurgery, St. Vincenz Hospital Paderborn, Am Busdorf 2, 33098 Paderborn, Germany; 6 Department of Neurosurgery, Marburg University Hospital, Baldingerstraße, 35043 Marburg, Germany

**Keywords:** KHVVG, minimum case volume thresholds, A2N40, neurosurgery, health-services-research

## Abstract

**Highlights:**

**What are the main findings?**

- At baseline (reporting year 2024), 66.6% of Germany’s area and 84.7% of the population (70.8 million people) were within a 40-min drive of an inpatient neurosurgical facility (A2N40).
- Each one-percentile-point increase in the simulated minimum case volume threshold reduced A2N40 area coverage by an average of 0.70 percentage points and left approximately 305,000 additional people without access, with the largest losses in rural regions of eastern Germany.

**What are the implications of the main findings?**

- Minimum case volume thresholds under the German Hospital Care Improvement Act create a measurable trade-off between centralisation and geographic accessibility, particularly in regions that are already underserved.
- The percentile-based simulation approach quantifies these geographic consequences before specific thresholds are legally defined and can therefore support evidence-based hospital planning.

**Abstract:**

**Background/Objectives:** The aim of this study was to simulate the impact of different minimum case volume thresholds, introduced under Germany’s Hospital Care Improvement Act, on the geographic accessibility of inpatient neurosurgical care. The Act introduces minimum case volume thresholds per service group, potentially leading to closures of low-volume neurosurgical units and reduced accessibility within 40 min of driving time (A2N40, Accessibility to a Neurosurgical Facility within 40 min). **Methods:** Inpatient case volumes of neurosurgical facilities were extracted from the Structured Quality Reports for reporting years 2023 and 2024, published by the Federal Joint Committee. Facilities were geocoded, 40-min driving time isochrones were generated, and the population within each isochrone was calculated. **Results:** At baseline in 2024, 66.6% of Germany’s area and 84.7% of the population were within the A2N40. Each one-percentile-point increase in the case volume threshold reduced A2N40 area coverage by an average of 0.70 percentage points, corresponding to approximately 305,000 additional people losing access, with the largest reductions observed in rural, less densely populated regions of the eastern federal states. **Conclusions:** Minimum case volume thresholds may substantially reduce the geographic accessibility of neurosurgical facilities, revealing a measurable trade-off between quality assurance through centralisation and geographic access to care, particularly in rural regions. The presented simulation approach can support evidence-based, geographically informed planning of future minimum case volume regulations.

## 1. Introduction

The relationship between hospital procedural volume and clinical outcomes has been described across numerous surgical disciplines, with higher-volume centres achieving lower operative mortality for complex procedures than lower-volume centres [1,2,3]. Building on this evidence, several health systems have pursued centralisation of specialised care at higher-volume centres [4]. While such concentration of care can improve clinical outcomes, it can simultaneously reduce geographic accessibility, particularly for populations in rural areas, as has previously been shown for critical care and neurosurgical services in other national health systems [5,6,7]. However, case volume alone does not determine hospital quality. Outcomes are also shaped by factors such as specialist availability, intensive care capacity, emergency readiness, equipment, referral pathways, transport systems, and case complexity. Centralisation strategies based primarily on minimum volume thresholds may therefore capture only part of what constitutes high-quality care.

Germany’s Hospital Care Improvement Act (in German ‘Krankenhausversorgungsverbesserungsgesetz’ or ‘KHVVG’) [8] represents one national implementation of this broader centralisation approach, and forms the basis of the present study. It introduces minimum case volume thresholds (known in Germany as ‘Mindestvorhaltezahlen’) per service group (known in Germany as ‘Leistungsgruppe’) to improve the quality of care by concentrating services at experienced centres [8,9]. For neurosurgery, among other fields, this could mean that facilities with insufficient case volumes may be closed in the future, as the hospital reform stipulates that no capacity-based payment (in German ‘Vorhaltevergütung’) is provided for the relevant service group if the minimum case volume thresholds are not met [8,10]. At the time of writing, no specific minimum case volume thresholds have yet been defined for neurosurgery. However, the simulation presented below anticipates the possible effects of such a stipulation. Rupa et al. [7] recently demonstrated that, given the current distribution of 182 neurosurgical departments, approximately 62.03% of Germany’s territory is covered by A2N40. Existing studies addressing the geographic accessibility of neurosurgical care in Germany, including Rupa et al. [7,11], have so far modelled accessibility under the current, static distribution of facilities, without simulating how accessibility would change if facilities were removed due to minimum case volume regulation. Conversely, studies evaluating minimum case volume standards, such as the MIVOS systematic review by Scharfe et al. [9], have focused on the relationship between case volume and clinical outcomes, without quantifying the resulting geographic consequences of facility closures. To the best of the authors’ knowledge, no study has yet combined case-volume-based facility exclusion with dynamic, nationwide geographic accessibility modelling to simulate the effects of minimum case volume thresholds before their concrete implementation. The present study addresses this gap by developing a percentile-based simulation approach that links facility-level case volume data directly to changes in geographic accessibility, allowing the consequences of a wide range of potential threshold levels to be quantified and visualised before any specific threshold for neurosurgery has been legally defined. We expected that increasing minimum case volume thresholds would reduce A2N40 coverage, particularly in rural areas. This article presents the simulation results of various graduated reductions in neurosurgical facilities on the A2N40.

## 2. Materials and Methods

### 2.1. Data Sources and Processing

The basis was the Structured Quality Reports (in German ‘Strukturierte Qualitaetsberichte’) in accordance with Section 136b German Social Code Book V (§136b SGB V) for the reporting years 2023 and 2024, published by the Federal Joint Committee (in German ‘Gemeinsamer Bundesausschuss, G-BA’) [12]. From the XML-formatted raw data, all entries with the department code (in German ‘Fachabteilungsschluessel’) 1700 (neurosurgery) were extracted. The following were recorded: site name, institutional classification number (in German ‘Institutskennzeichen’), campus identification number (in German ‘Standortnummer’), address, and numbers of inpatient and day-case cases. Two facilities were excluded; although they had reported the department code 1700, they reported an inpatient and day-case case count of 0. These two facilities were manually verified via their official hospital websites and subsequently excluded, as no neurosurgical care was provided at either site.

The accessibility analysis follows a four-stage pipeline:

(1) Geocoding of facility addresses;
(2) Generation of 40-min driving-time isochrones;
(3) Precomputed point-in-polygon assignment of census grid cells to facility isochrones;
(4) Real-time population and area aggregation based on the active facility set.

The web application accessible at www.neurochirurgie-atlas.de serves as an implementation and visualisation layer for this pipeline. The web application was developed using AI-assisted code generation, a practice referred to as ‘vibe coding’ in the cited literature [13], with the use of Claude Sonnet 4.6 and Opus 4.7 (Anthropic PBC, San Francisco, CA, USA) [14] in an iterative, conversation-based process. The AI was provided with the clinical and methodological context. It generated the application code, including the map interface, filter logic and population calculation. Architectural decisions, data validation and technical accuracy remained the responsibility of the working group. The underlying methodology is described in full in the Supplementary Text S1. The complete source code of the pipeline is publicly archived at Zenodo [15].

The minimum case volume threshold filter allows the specification of a percentile threshold (0–100%) representing the cumulative proportion of the total case volume to be excluded. Facilities are sorted by descending inpatient case count and removed sequentially, starting with the lowest case count, until the cumulative volume reaches the threshold. Removed facilities disappear from the map display with isochrone overlay and from the coverage calculation.

The percentile-based simulation approach is directly grounded in enacted German law, which mandates that the IQWiG (Institute for Quality and Efficiency in Health Care) develop recommendations for minimum case volume thresholds “in the form of a percentile of the number of all treatment cases of a calendar year in which services from the respective service group were provided” [16]. Rather than assuming a specific absolute threshold, which requires IQWiG recommendations and subsequent regulatory decisions not yet available at the time of writing, the percentile approach models the full range of plausible threshold scenarios in a form that is both policy-consistent and directly comparable to the regulatory instrument once it is applied. The simulation range of 0–20% was selected to reflect plausible policy scenarios based on the distributional structure of neurosurgical case volumes across the 200 facilities included for 2024, encompassing scenarios from marginal concentration to substantial restructuring of the neurosurgical landscape. This range was chosen to cover the spectrum of case-volume exclusion resulting from the underlying legislative thresholds, consistent with the study’s aim of modeling the consequences of the legislative framework itself rather than deriving an independent empirical optimum.

To assess data quality and internal consistency, a random sample of 20 of the 200 included facilities (10%) was manually validated against the source data. For each sampled facility, the reported case volume, the geocoded location, and the population figure within the 40-min isochrone were manually cross-checked. In addition, the ranking of all facilities by case volume and the resulting number of excluded facilities at the 5%, 10%, 15%, and 20% percentile thresholds were manually recalculated and verified against the simulation output. All values were found to be correct and plausible.

### 2.2. Statistical Analysis

The relationship between the percentile threshold and A2N40 area coverage or the unserved population was assessed across 21 discrete simulation points, corresponding to percentile thresholds from 0% to 20% in increments of 1 percentage point. Each point represents a single deterministic output of the simulation rather than an observation drawn from a sampled population, so the resulting dataset constitutes a fully enumerated functional relationship rather than empirical data in the conventional sense. To summarise this relationship in compact form, ordinary least squares linear regression was fitted to the 21 threshold-outcome pairs, and the slope (β) and coefficient of determination (R^2^) are reported descriptively, characterising the average rate of change and the extent to which a single straight line approximates the simulated pattern. As no sampling error exists for deterministic model output, the regression is not used, and should not be interpreted, as a formal hypothesis test, and *p*-values are therefore not reported. The interval reported alongside the slope reflects the range of average segment-wise slopes calculated across four equal five-percentile-point sub-ranges of the simulated series (0–5%, 5–10%, 10–15%, 15–20%; Table 1), rather than a confidence interval in the inferential statistical sense, and is included to illustrate that the underlying dose–response relationship is not perfectly linear.

## 3. Results

**Figure 1:**
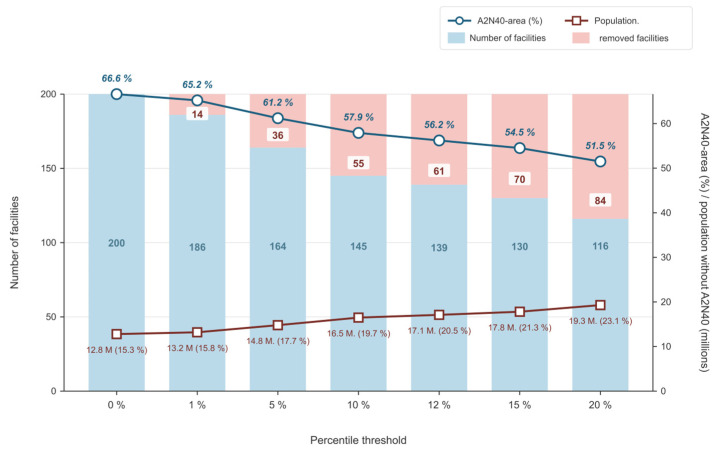
Impact of rising percentile thresholds on neurosurgical coverage (reporting year 2024). Blue line: A2N40 area coverage (%); red line: population without A2N40 (millions); bars: remaining (blue) and removed (red) facilities. Own representation.

The simulation reveals a disproportionately steep decline in accessibility even at low exclusion thresholds, with the marginal loss of coverage per percentile point decreasing as the threshold increases. A total of 197 neurosurgical facilities with valid data for 2023 and 200 for 2024 were identified. Total case volume: 214,891 inpatient cases in 2023 and 225,747 inpatient cases for 2024. At baseline (0% percentile), 70.8 million (84.7%) of the population had access to a neurosurgical facility within a 40-min drive (A2N40), whilst 12.8 million (15.3%) were located outside a 40-min catchment area. As the percentile value increased, population coverage declined (Figure 1). At a 20% threshold, 19.3 million people (23%) lived outside the A2N40, compared with 12.8 million (15.3%) at baseline—an increase of 6.5 million people. Rather than following a constant linear trend, the marginal loss of A2N40 area coverage decreases progressively across the simulated threshold range, from −1.08 percentage points per percentile point in the 0–5% range to −0.66, −0.68, and −0.60 percentage points in the 5–10%, 10–15%, and 15–20% ranges, respectively (Table 1). This pattern indicates a concave dose–response relationship, in which the earliest facility closures, affecting the lowest-volume and often geographically isolated units, have a disproportionately large impact on accessibility, while subsequent closures increasingly affect facilities whose catchment areas already overlap with neighbouring institutions. The single linear regression coefficient describing the overall trend across the full 21-point simulation series shown in Figure 1 is β = −0.70 percentage points per percentile point (R^2^ = 0.980, range of segment-wise slopes: −1.08 to −0.60), and is reported as a compact summary of the overall pattern rather than evidence of a constant marginal effect. Similarly, the unserved population increases on average by 0.30 million per percentile point (R^2^ = 0.982), corresponding to approximately 305,000 additional people without A2N40 access per percentile point.

**Figure 2:**
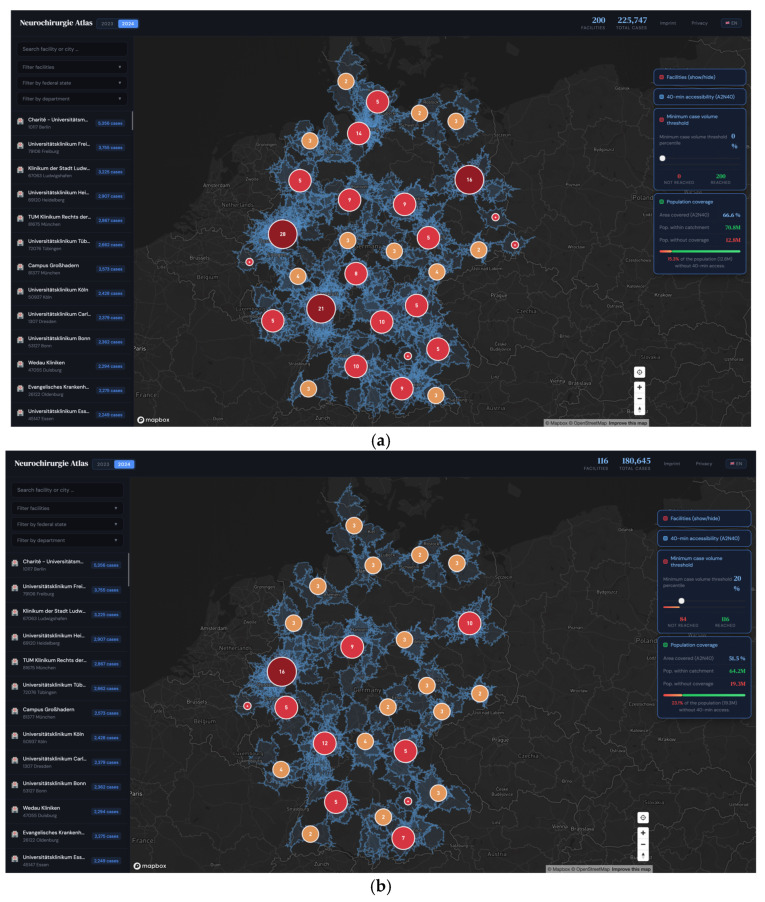
Screenshots of the simulation tool (neurochirurgie-atlas.de, 2024 data) showing 40-min accessibility of neurosurgical facilities in Germany (A2N40). Blue areas denote the merged 40-min isochrones, red circles denote active facilities (clustered markers show the number of aggregated facilities), the left-hand panel serves for filtering and the right-hand panel reports population and area coverage. (**a**) No minimum case volume threshold (0th percentile, 200 facilities). (**b**) Threshold at the 20th percentile (116 facilities).

The impact of rising thresholds is not spatially uniform: losses concentrate first in the already underserved eastern federal states before extending to peripheral regions further west. Assuming that neurosurgical units are closed if the minimum case volume thresholds are not met and no new units are established, increasing thresholds lead to growing gaps in care, initially in rural regions of the new federal states, and then spreading to peripheral areas of Lower Saxony, Bavaria and Baden-Württemberg. A 5% exclusion removes 36 facilities with 5.4 percentage points less A2N40, whilst a 20% exclusion removes 84 facilities with 15.1 percentage points less A2N40 area (Figure 1 and Figure 2). In a year-on-year comparison between 2023 and 2024, the Structured Quality Reports for 2024 include 3 more facilities (200 vs. 197 departments). No significant differences are apparent between the data years. The geographical impact of identical thresholds was comparable in both years.

## 4. Discussion

The analysis makes the potential conflict of objectives between the reform instruments of the KHVVG, aspects of quality assurance through concentration and specialization on the one hand, and security of care as well as geographical accessibility on the other, visible and quantifiable. Therefore the simulation models a static, worst-case scenario in which facilities below the threshold cease operation without compensatory adaptation. It does not evaluate clinical quality, mortality, complications, costs, or capacity, and it does not identify an optimal case volume threshold.

The simulation shows that even moderate minimum case volume thresholds can substantially reduce coverage, particularly in the new, eastern federal states, where significantly lower A2N40 figures have already been identified [7]. This creates a tension between quality assurance through minimum case volume thresholds and maintaining accessibility, for which solutions are required.

The clinical relevance of reduced neurosurgical accessibility is established in the literature. Peterman et al. demonstrated that traumatic brain injury (TBI) mortality within a county increases by 10% for every 25 miles of additional distance to the nearest neurosurgeon [17], directly quantifying the outcome penalty associated with geographic inaccessibility. The same study noted that patients with cerebral aneurysms living farther from neurosurgical care are more likely to undergo emergent treatment after rupture rather than elective repair, a finding with direct implications for preventable morbidity and mortality. In the German context, the simulation presented here shows that a 20% percentile threshold removes 84 facilities and leaves an additional 6.5 million people outside A2N40, a population increase of approximately 50% relative to baseline. The present study did not evaluate clinical outcomes, mortality, or patient prognosis, and the distance–mortality relationship reported by Peterman et al. was derived from a different healthcare system (the United States) with distinct emergency care structures, insurance-based access barriers, and population distribution. Direct quantitative extrapolation of this relationship to the German setting is therefore not warranted. Nonetheless, it is plausible to hypothesise that the accessibility losses documented here could be associated with a comparable, though as yet unquantified, clinical outcome burden, disproportionately affecting already underserved rural populations in eastern Germany, Lower Saxony, and peripheral Bavaria. Testing this hypothesis directly in the German context remains an important direction for future research.

### 4.1. Practical Implications

The following measures are not themselves evaluated within the present simulation framework but are discussed as potential strategies for mitigating the modelled accessibility losses, informed by the broader literature on care network design.

One possible way to mitigate this loss of accessibility could lie in the establishment of neurosurgical ‘satellite sites’. These would be smaller facilities with a reduced but defined range of services, which could in particular ensure neurotraumatological care and selected spinal surgical procedures, whilst complex procedures would continue to be performed at supra-regional centres.

As part of the upcoming hospital reform, the service group “29—Spinal surgery” may be allocated nationwide. This is managed separately from service group “52—Neurosurgery” [18]. Provided that the spinal surgery service group is staffed by neurosurgeons and that a defined range of procedures may be performed within it, for example, not limited to emergency neurotraumatological procedures on the skull and brain, this could serve as an additional incentive to maintain a neurosurgical presence at smaller sites.

In order to attract neurosurgeons to work at less attractive locations, further incentives must be created in addition to a sufficiently attractive range of potential cases. A rotation programme in cooperation between a larger and a smaller unit, in which specialists are deployed to peripheral locations for a limited period, could be one such measure. When establishing a department for general neurosurgery as well as for specialised spinal surgery, ensuring the availability of particularly experienced specialist backup for a certain period, for example, can be helpful.

Specialisation and centralisation lead to an increasing demand for transport capacity between hospitals [19,20]. At the same time, several studies show that expanding the helicopter rescue infrastructure can significantly improve access to specialised care in sparsely populated regions [6,11,21,22]. Ernstberger et al. [21] were also able to demonstrate that, within a well-developed trauma network, the mortality rate of seriously injured patients is independent of the mode of transport and the level of care provided by the receiving hospital, which speaks to the robustness of network-based care structures. The creation and expansion of ground- and air-based transport capacities can therefore make a significant contribution to ensuring the quality of care within the context of the centralisation and specialisation of medical services.

The mitigation strategies outlined above—satellite sites, rotation systems, and expanded transport infrastructure—represent system adaptations that would, if implemented, partially offset the accessibility losses modelled here. To contextualise the magnitude of this offset, it is instructive to consider three stylised scenarios of system response to facility closure. In the first scenario (static closure, corresponding to the present model), all removed facilities cease operation with no compensatory adaptation. This represents a deliberate worst-case assumption that isolates the pure geographic effect of threshold-driven concentration and serves as an upper bound of accessibility loss. In the second scenario (partial substitution), patients previously served by a removed facility are absorbed by the nearest remaining active facility: in regions where isochrone overlap already exists between neighbouring facilities, a proportion of the affected population retains A2N40 access without additional infrastructure, an effect that is implicitly present in the precomputed coverage data but not separately quantified here. In regions without isochrone overlap, precisely those rural areas identified as most vulnerable, substitution is structurally impossible without new infrastructure. In the third scenario (transport augmentation), expanded air-based emergency transport extends effective reach beyond the A2N40 boundary, consistent with the helicopter rescue infrastructure described by Ernstberger et al. [21] and Hoechter et al. [22]. Under this scenario, a proportion of the newly unserved population could regain effective accessibility, though at higher system cost and with reduced reliability under adverse weather conditions. Across all three scenarios, the spatial pattern of vulnerability remains consistent: rural regions in eastern Germany, peripheral Lower Saxony, and southern Bavaria are disproportionately affected regardless of the degree of system adaptation, because they combine low baseline facility density with limited transport infrastructure and the greatest isochrone gaps. The present simulation quantifies the outer bound of this vulnerability. The actual impact under the KHVVG will depend on the speed and completeness of adaptive system responses, which lie beyond the scope of this analysis to predict.

### 4.2. Limitations

At the time of writing, the methodology of the IQWiG for determining minimum case volume thresholds is known [23], but specific minimum case volume thresholds or percentiles for the neurosurgery service group are not. However, they are generally envisaged for the service groups. In this analysis, therefore, percentile values within the range of 0 to 20%, measured against the total volume of neurosurgical cases, are used.

The case volumes used in this simulation are extracted from the Structured Quality Reports at the level of the organisational unit with department code 1700 (neurosurgery), which captures all inpatient cases attributed to the neurosurgical department as a whole. Minimum case volume thresholds, however, are defined per service group, not per department. The neurosurgical service groups most relevant to this reform are LG 52 (Neurosurgery) and LG 29 (Spinal Surgery), both of which may be provided by the same departmental unit, so the service-group-specific case volumes attributable to LG 52 alone cannot be derived from the Structured Quality Reports. This mismatch introduces uncertainty at two levels. First, the absolute case volumes used in this simulation are likely to overestimate the LG-52-specific volumes, because they include cases that would be attributed to other service groups (e.g., LG 29). Second, for the 24 facilities in the 2024 dataset that reported more than one department code alongside code 1700, the reported case counts may additionally include non-neurosurgical treatment cases, further inflating the absolute volumes for those facilities.

However, the impact of this overestimation on the relative ranking of facilities, which determines the order of simulated closure, is expected to be limited in the following direction: if LG-52-specific volumes are proportionally distributed across all facilities (i.e., the share of LG-52 cases is approximately constant across facilities of different sizes), the rank order is preserved and the simulation remains qualitatively valid. Systematic bias would arise only if small-volume facilities disproportionately contribute non-LG-52 cases, for example, if low-volume departments predominantly treat spinal surgery cases rather than intracranial neurosurgery. In that scenario, the simulation would underestimate the true threshold at which small facilities would be affected, meaning the actual geographic impact of minimum case volume requirements could be more pronounced than reported. Conversely, if high-volume facilities have a higher share of non-LG-52 procedures, the simulation would overestimate the impact. In the absence of service-group-specific case data, neither direction can be excluded. The simulation should therefore be interpreted as modelling the effect of case-volume-based concentration on geographic accessibility under a structural equivalence assumption, rather than as a direct operationalisation of LG-52 minimum case volume thresholds.

The web application is based on data from the structured quality reports rather than on information from the federal states’ hospital plans, which explains the difference in the number of institutions and the baseline values for A2N40 (66.6% versus 62.03%) between the current study and Rupa et al. [7].

The content-related validity of the model was assessed through manual spot-checking of a 10% random sample of facilities, covering case volume, geocoding, and population calculation. While this manual spot-check did not reveal discrepancies in the sampled facilities, it does not constitute a systematic, independent technical validation of the underlying geoprocessing pipeline, such as cross-validation of isochrone geometries or population aggregation against an established, independent GIS software package (e.g., QGIS or OpenRouteService). Subtle, systematic errors affecting facilities outside the sampled subset, or algorithmic biases not detectable through manual case-by-case review, cannot therefore be fully excluded.

Simplifications in the individual steps are necessary for implementation as a dynamic, nationwide overall simulation. Locally limited assessments of the effects on individual neurosurgical facilities should therefore be verified and validated on a case-by-case basis.

The travel time isochrones for A2N40 are based on the standard routing algorithm of the Mapbox Isochrone API (v3.3.0) and represent typical travel times without taking into account fluctuations depending on the time of day, traffic conditions, or weather conditions. The model is further limited to automobile travel time and does not account for public transportation availability, which may represent the only feasible mode of access for parts of the population. Furthermore, neither cross-border (international) care or helicopter-supported emergency services, nor receiving-hospital capacity, waiting times, referral pathways, or the clinical urgency of individual neurosurgical conditions were modelled.

A further limitation concerns the AI-assisted development of the web application. A total of 3900 lines of code were generated using large language models, enabling the implementation of a technically complex, nationally scaled GIS simulation without requiring professional software development expertise. However, the validity of AI-generated code cannot be verified through code review alone in the conventional sense. Rather, it can only be assessed indirectly through the plausibility and consistency of the simulation outputs. This shifts the burden of quality assurance from writing code to reviewing, interpreting and validating results, a subtle but important distinction. Furthermore, particularly for non-professional developers, AI-generated codebases retain an element of opacity: while the functional output may be verifiable, the underlying logic of individual code segments may not be fully transparent or comprehensible to the implementing author. This represents a form of methodological black box that should be acknowledged when AI-assisted programming is used in scientific contexts.

## 5. Conclusions

Minimum case volume thresholds under the KHVVG have the potential to significantly reduce the geographical accessibility of inpatient neurosurgical facilities, particularly in regions that are already underserved. Legislative interventions, as well as hospital planning decisions by the federal states and the setting of minimum case volume thresholds by the IQWiG, should be based on dynamic impact analyses. The simulation method presented here is such a web-based simulation tool, based on publicly available data for simulating the effect of minimum case volume thresholds. Minimum volumes (in German ‘Mindestmengen’) for defined procedures pursuant to Section 136b of the German Social Code Book V have not (currently) been announced for neurosurgery [24]. The simulation can be adapted to specific questions, e.g., minimum volumes for defined procedures.

Beyond its application to neurosurgery, the principal contribution of this work lies in the simulation methodology itself, a transferable, percentile-based approach linking facility-level case volume data to dynamic geographic accessibility modelling, which could support evidence-based healthcare planning in advance of policy implementation; validating this approach in other healthcare systems and medical specialties represents an important direction for future research. Beyond this methodological transfer, the framework itself should be extended by incorporating clinical outcomes and health-related endpoints, economic evaluations of centralisation versus decentralised care, and more sophisticated models of healthcare reorganisation that account for dynamic system adaptation, in order to enable a more comprehensive assessment of the true impact of centralising neurosurgical services.

## Figures and Tables

**Table 1:** Segment-wise average marginal decrease in A2N40 area coverage across the simulated percentile threshold range (0–20%), based on the 21-point simulation series.

Threshold Range	ΔA2N40 Area (pp)	Steps	Ø per Percentile Point (pp)
0–5%	−5.4	5	−1.08
5–10%	−3.3	5	−0.66
10–15%	−3.4	5	−0.68
15–20%	−3.0	5	−0.60

## Data Availability

The data presented in this study are openly accessible via the web application at https://www.neurochirurgie-atlas.de (accessed on 4 August 2026). The complete source code of the data processing pipeline and web application is archived on Zenodo at https://doi.org/10.5281/zenodo.20520666 (version 1.0.0) (accessed on 4 August 2026). The underlying source data (Structured Quality Reports) are publicly available from the Federal Joint Committee (G-BA) at https://www.g-ba.de (accessed on 5 July 2026). Census population data are available from Destatis at https://www.zensus2022.de (accessed on 5 July 2026).

## Supplementary Materials

The following supporting information can be downloaded at: https://www.mdpi.com/article/10.3390/healthcare14152437/s1. Table S1: Simulated effects of minimum case volume thresholds (0–20%, in 1-percentage-point increments) on the number of neurosurgical facilities, A2N40 area coverage, and population without A2N40 access (reporting year 2024). Figure S1: Complete simulated curve of A2N40 area coverage (%, solid line, circles) and population without A2N40 (millions, dashed line, squares) across all 21 simulated percentile threshold points (0–20%, in 1-percentage-point increments). Own representation. Text S1: Description of the complete data sourcing and processing. References [7,12,13,14,15,16,25,26,27,28,29,30,31,32,33,34,35,36,37,38,39,40] are cited in the Supplementary Materials.

## Abbreviations

The following abbreviations are used in this manuscript:

A2N40	Accessibility of a Neurosurgical Facility within a 40-min Drive
KHVVG	Krankenhausversorgungsverbesserungsgesetz [Hospital Care Improvement Act]
G-BA	Gemeinsamer Bundesausschuss [Joint Federal Committee]
SGB V	Sozialgesetzbuch, Buch V [German Social Code, Book V]
TBI	Traumatic Brain Injury
WGS 84	World Geodetic System 1984
INKAR	Indikatoren und Karten zur Raum- und Stadtentwicklung [Indicators and Maps on Spatial and Urban Development]
IQWiG	Institut für Qualität und Wirtschaftlichkeit im Gesundheitswesen [Institute for Quality and Efficiency in Health Care]
