# Supplementary material for: Simulation of the Effects of Minimum Case Volume Thresholds on the Geographic Accessibility of Inpatient Neurosurgical Facilities in Germany
Source: Healthcare (Basel). 2026 Aug 6;14(15):2437. doi: 10.3390/healthcare14152437 (PMC13465607; doi:10.3390/healthcare14152437)
Supplement: Supplementary file 1 [file healthcare-14-02437-s001.zip › healthcare-4365685-supplementary.pdf]

healthcare

Supplementary Materials

Table S1. Simulated effects of minimum case volume thresholds (0-20%, in 1-percentage-point increments) on the number of neurosurgical facilities, A2N40 area coverage, and population without A2N40 access (reporting year 2024).

Percentile threshold (%)	Facilities removed (n)	Facilities remaining (n)	A2N40 area coverage (%)	Population without A2N40 (millions)	Population without A2N40 (%)
0	0	200	66.6	12.8	15.3
1	14	186	65.2	13.2	15.8
2	21	179	63.9	13.6	16.3
3	27	173	63.3	14.0	16.7
4	32	168	61.6	14.7	17.6
5	36	164	61.2	14.8	17.7
6	41	159	59.9	15.3	18.3
7	44	156	59.8	15.4	18.4
8	48	152	59.1	15.7	18.8
9	52	148	58.8	15.8	18.9
10	55	145	57.9	16.5	19.7
11	59	141	56.3	17.1	20.5
12	61	139	56.2	17.1	20.5
13	64	136	55.9	17.2	20.6
14	67	133	55.2	17.5	20.9
15	70	130	54.5	17.8	21.3
16	73	127	53.5	18.1	21.7
17	75	125	53.4	18.2	21.8
18	78	122	53.4	18.2	21.8
19	81	119	52.3	18.7	22.4
20	84	116	51.5	19.3	23.1

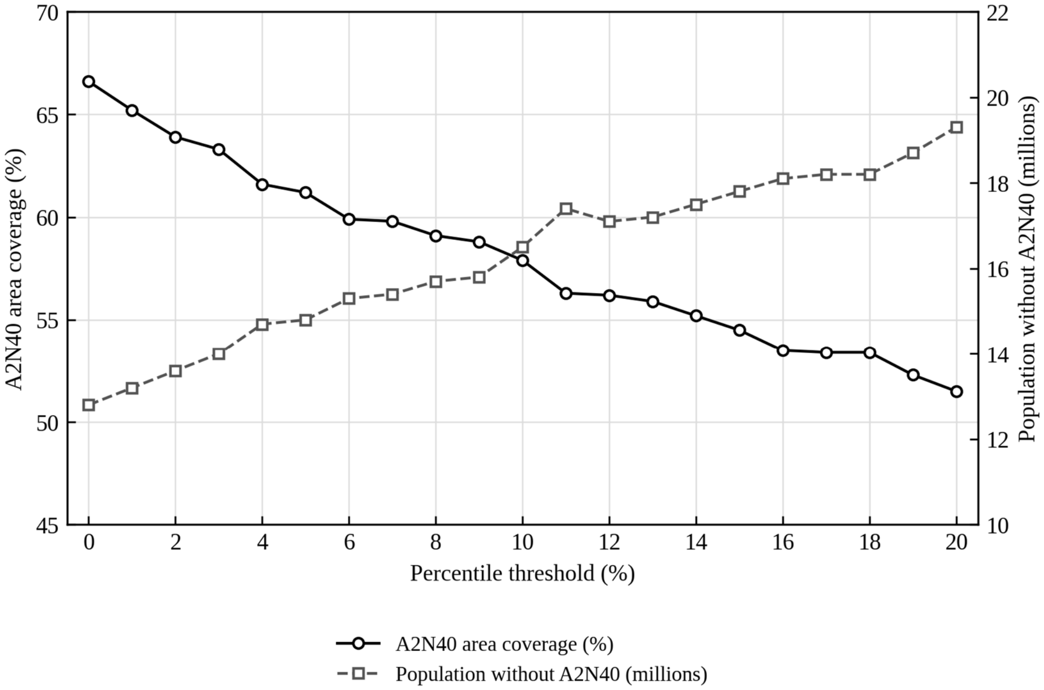

Figure S1. Complete simulated curve of A2N40 area coverage (%, solid line, circles) and population without A2N40 (millions, dashed line, squares) across all 21 simulated percentile threshold points (0-20%, in 1-percentage-point increments). Own representation.

Supplementary Text S1: Description of the complete Data sourcing and processing

Data sources and processing

The basis was the Structured Quality Reports (in German 'Strukturierte Qualitaets-berichte') in accordance with Section 136b German Social Code Book V (§136b SGB V) for the reporting years 2023 and 2024, published by the Federal Joint Committee (in German 'Gemeinsamer Bundesausschuss, G-BA') [12]. From the XML-formatted raw data, all entries with the department code (in German 'Fachabteilungsschluessel') 1700 (neurosurgery) were extracted using an automated JavaScript script (fast-xml-parser (v 5.3.7) [25]). The following were recorded: site name, institutional classification number (in German 'Institutskennzeichen'), campus identification number (in German 'Standortnummer'), address, and numbers of inpatient and day-case cases. Two facilities were excluded, although they had reported the department code 1700, they reported an inpatient and day-case case count of 0. The facility addresses were geocoded using the Mapbox (Mapbox Inc., San Francisco, CA, USA) Geocoding API [26] with a fallback to the OpenStreetMap (OpenStreetMap Foundation, Cambridge, UK) Nominatim API [27]. Ambiguous results were manually verified via Google Maps (Google LLC, Mountain View, CA, USA) [28]. The assignment to the federal state was carried out automatically using postcodes based on the municipal directory data from the Federal Statistical Office [29].

Isochrones and population data

For each geocoded facility, 40-minute travel time isochrones were retrieved via the Mapbox Isochrone API [26]. The isochrone defines the polygonal area from which a facility can be reached by car within 40 minutes. The geometries were stored as GeoJSON polygons in a Supabase (Supabase Inc., San Francisco, CA, USA) database [30].

The population per isochrone was calculated using the population grid from the 2022 census [31] (resolution 1 km², EPSG projection: 3035). The population served was determined on the basis of the pre-calculated assignment table: for each census cell, a check is made to see whether at least one of the active (i.e. not removed by the case count filter) facilities covers the cell. The population served is calculated as the sum of the population figures for all cells covered in this way. The unserved population is calculated as the difference from the total INKAR population [32]. The area-based coverage (A2N40 area) was calculated using grid sampling (Turf.js (v7) [33], 1-km grid): The union area A-union is calculated as the number of covered grid points multiplied by the cell area, limited to 357,684 km². This separate calculation is necessary because the census grid contains only inhabited cells and therefore does not directly reflect the total area within the isochrones (including uninhabited areas such as forests and water bodies).

The grid cell centres were transformed precisely to the World Geodetic System 1984 (WGS 84) [34] using the JavaScript library "proj4" v 2.20.3 [35]. In order to be able to calculate the population within all active isochrones (= served population) in real time within the web application, the computationally intensive geometry calculation was pre-calculated once on the server side. Using a two-stage point-in-polygon test (bounding box prefilter [36], followed by ray casting [37]), it was determined for each of the approximately 210,000 inhabited census cells which isochrones they cover. The result is a mapping table stored in the database, which contains the population figure and the list of associated campus identification number for each cell. Subsequently, a simple comparison of the visible campus identification number is sufficient, without the need for further geometric operations. The data from the INKAR database (Indicators and Maps on Spatial and Urban Development) [32] served as a reference for the total population: 83,577,140 inhabitants across 357,684 km². This reference data is necessary for standardisation, as the sum of the 2022 census population data does not correspond exactly to the official total population due to rounding effects and uninhabited grid cells.

The 40-minute driving time threshold defining A2N40 was selected on two complementary grounds. First, it was adopted from Rupa et al. [7], who established this threshold as the methodological baseline for quantifying neurosurgical accessibility in Germany, enabling direct comparability of the baseline results presented here with that prior work. Second, the 40-minute threshold is grounded in the legislative process of the KHVVG itself: the ministerial draft of 15 April 2024 explicitly specified a maximum driving time of 40 min as the accessibility criterion for all service groups other than general internal medicine and general surgery, stating that both travel time and the number of residents affected by longer travel times should be considered in assessing area-wide accessibility [38]. Although this provision was removed from the final enacted legislation, it reflects the legislator's own operational definition of acceptable geographic reach for specialised inpatient services at the time of the reform's design. The question of how this accessibility threshold relates to the quality of neurosurgical care is scientifically highly relevant and will be the subject of further analyses in forthcoming work.

Minimum case volume threshold filter and population coverage

The minimum case volume threshold filter allows the specification of a percentile threshold (0–100%) representing the cumulative proportion of the total case volume to be excluded. Facilities are sorted by descending inpatient case count and removed sequentially, starting with the lowest case count, until the cumulative volume reaches the threshold. Removed facilities disappear from the map display with isochrone overlay and from the coverage calculation. The population served was determined on the basis of the pre-calculated assignment table: for each census cell, a check is made to see whether at least one of the active (i.e. not removed by the case count filter) facilities covers the cell. The

population served is calculated as the sum of the population figures for all cells covered in this way. The unserved population is calculated as the difference from the total INKAR population [32]. The area-based coverage (A2N40 area) was calculated using grid sampling (Turf.js [33], 1-km grid): The union area A-union is calculated as the number of covered grid points multiplied by the cell area, limited to 357,684 km². This separate calculation is necessary because the census grid contains only inhabited cells and therefore does not directly reflect the total area within the isochrones (including uninhabited areas such as forests and water bodies).

The percentile-based simulation approach is directly grounded in enacted German law. Section 135f(2) of the German Code Book V, as introduced by the KHVVG, explicitly mandates that the IQWiG (Institute for Quality and Efficiency in Health Care) develop recommendations for minimum case volume thresholds "in the form of a percentile of the number of all treatment cases of a calendar year in which services from the respective service group were provided" [16]. Rather than assuming a specific absolute threshold, which requires IQWiG recommendations and subsequent regulatory decisions not yet available at the time of writing, the percentile approach models the full range of plausible threshold scenarios in a form that is both policy-consistent and directly comparable to the regulatory instrument once it is applied. The simulation range of 0–20% was selected to reflect plausible policy scenarios based on the distributional structure of neurosurgical case volumes across the 200 facilities included for 2024, encompassing scenarios from marginal concentration to substantial restructuring of the neurosurgical landscape.

Accessibility analysis

The accessibility analysis follows a four-stage pipeline: (1) geocoding of facility addresses, (2) generation of 40-minute driving-time isochrones, (3) precomputed point-in-polygon assignment of census grid cells to facility isochrones, and (4) real-time population and area aggregation based on the active facility set. The web application accessible at www.neurochirurgie-atlas.de serves as an implementation and visualisation layer for this pipeline. The complete source code of the pipeline is publicly archived at Zenodo [15].

AI-supported development

The web application (Node.js/Express v 4.22.1 [39,40], Mapbox GL JS [26], Supabase-js (v 2.98.0) [30]) was developed using AI-assisted programming via 'Vibe Coding' [13] with the use of Claude Sonnet 4.6 and Opus 4.7 (Anthropic PBC, San Francisco, CA, USA) [14] in an iterative, conversation-based process. The AI was provided with the clinical and methodological context. It generated the application code, including the map interface, filter logic and population calculation. Architectural decisions, data validation and technical accuracy remained the responsibility of the working group.

https://doi.org/10.3390/healthcare14152437
